# Cytokines are the Basis of the Development and Suppression of Inflammation in Atherosclerosis

**DOI:** 10.31083/RCM26421

**Published:** 2025-03-12

**Authors:** Alexander V. Blagov, Alexey V. Churov, Irina A. Starodubtseva, Tatiana I. Kovyanova, Tamara B. Pecherina, Vasily N. Sukhorukov

**Affiliations:** ^1^Institute of General Pathology and Pathophysiology, 125315 Moscow, Russia; ^2^Institute on Aging Research, Russian Gerontology Clinical Research Centre, Pirogov Russian National Research Medical University, 129226 Moscow, Russia; ^3^Department of Polyclinic Therapy, NN Burdenko Voronezh State Medical University, 394036 Voronezh, Russia; ^4^Institute for Atherosclerosis Research, 121609 Moscow, Russia; ^5^Laboratory for Cardiac Fibrosis, Research Institute for Complex Issues of Cardiovascular Diseases, 650002 Kemerovo, Russia; ^6^Institute of Experimental Cardiology, Russian Medical Research Center of Cardiology, 121552 Moscow, Russia

**Keywords:** atherosclerosis, cytokines, cytokine-targeted therapy, cytokines in atherosclerosis

## Abstract

Cardiovascular diseases continue to be the primary cause of mortality in industrialised countries, and atherosclerosis plays a role in their development. A persistent inflammatory condition affecting big and medium-sized arteries is known as atherosclerosis. It is brought on by dyslipidemia and is facilitated by the immune system’s innate and adaptive components. At every stage of the progression of atherosclerosis, inflammation plays a crucial role. It has been demonstrated that soluble factors, or cytokines, activate cells involved in the pathophysiology of atherosclerosis and have a significant impact on disease progression. Anti-inflammatory cytokines (such as interleukin (IL)-5 and IL-13) mitigate atherosclerosis, whereas pro-inflammatory cytokines (such as IL-1, IL-6) quicken the disease’s course. Of interest is the fact that a number of cytokines can exhibit both atherogenic and atheroprotective properties, which is the topic of study and discussion in this review. This review provides a comparative analysis of the functions of the main cytokines involved in the pathogenesis of atherosclerosis. Their functional relationships with each other are also shown. In addition, potential therapeutic strategies targeting these cytokines for the treatment of atherosclerosis are proposed, with an emphasis on recent clinical research in this area.

## 1. Introduction

Atherosclerosis is a chronic inflammatory disease of the arteries that accounts for about 50% of all deaths in Western society. It is primarily a lipid process initiated by the accumulation of low-density lipoproteins and residual lipoprotein particles, as well as an active inflammatory process in focal areas of the arteries, especially in areas of impaired nonlaminar flow at arterial branch points. It is considered a major cause of the occurrence of atherosclerotic cardiovascular disease (ASCVD) leading to heart attacks, strokes and peripheral artery disease [1].

Because atherosclerosis is a predominantly asymptomatic condition, it is difficult to accurately determine the incidence. Atherosclerosis is considered the main cause of cardiovascular disease. Atherosclerotic cardiovascular diseases mainly affect the heart and brain: coronary heart disease (CHD) and ischemic stroke. Ischemic heart disease (IHD) and stroke are the first and fifth causes of death in the world, respectively [2].

Studies in humans and animals have established that atherosclerosis is caused by a chronic inflammatory process in the arterial wall, initiated primarily in response to endogenously altered structures, in particular oxidized lipoproteins, which stimulate both innate and adaptive immune responses [3]. The innate response is initiated by the activation of both vascular cells and monocytes/macrophages. Subsequently, the adaptive immune response develops against a variety of potential antigens presented to effector T lymphocytes by antigen-presenting cells. Vascular cells, endothelial cells and smooth muscle cells (SMCs) are involved in disease progression by providing feedback to maintain inflammation through the release of pro-inflammatory cytokines and chemokines. Cytokines play a dual role in atherosclerosis. Pro-inflammatory cytokines promote disease development and progression, whereas anti-inflammatory and T-cell-associated regulatory cytokines exert distinct antiatherogenic activity [4].

There is currently a growing interest in a new therapeutic area, namely agents directed against specific targets in the inflammatory cascade. New strategies are developing in the battle against atherosclerosis, and several of them show promise as our understanding of the function of cytokines in atherogenesis grows. The first objective of this review is to compare the functions of different types of cytokines in the pathogenesis of atherosclerosis, using interleukin (IL)-6 as an example, show in detail the ability of some cytokines to exhibit both atherogenic and atheroprotective properties. The second objective of this review is to select the most promising cytokines as therapeutic targets and to analyze existing preclinical and clinical studies using them.

## 2. Pro-Inflammatory Cytokines in the Pathogenesis of Atherosclerosis

### 2.1 Interleukin-6 (IL-6)

IL-6 is a significant cytokine implicated in several cardiac conditions. IL-6 possesses pro-inflammatory characteristics and can also exhibit anti-inflammatory features. IL-6 is generated by fibroblasts, endothelial cells, macrophages, monocytes, and vascular smooth muscle cells (VSMCs) in cardiovascular disorders [5]. Oxidized low density lipoproteins (oxLDL) which are formed in atherosclerosis initiate toll-like receptors (TLR especially TLR2/4) activation. It causes activation of the NOD-like receptor protein 3 (NLRP3) inflammasome and further maturation of IL-1 and IL-18 cytokines which stimulate myeloid cells to release IL-6 in addition to tumor necrosis factor alpha (TNF-α) [6]. This quickly increases the synthesis of IL-6 and starts a large positive feedback loop [7].

To initiate the conventional signaling cascade, IL-6 attaches to the membrane-bound (mb) IL-6 receptor (IL-6R) (mbIL-6R), which is found on some types of leukocytes. Intracellular signaling pathways, such as the Janus kinase/signal transducer and activator of transcription (JAK/STAT) pathway, are dimerized and activated by the IL-6/IL-6R complex [5]. Since soluble IL-6R (sIL-6R) enables IL-6 to stimulate cells lacking IL-6R on their membranes, it is crucial for trans-signaling. While trans-activation is linked to pro-inflammatory effects, classic IL-6 signaling is thought to have anti-inflammatory qualities [7]. The process of IL-6 signaling begins with the binding of the IL-6/IL-6R complex to the gp 130 protein. The newly formed complex activates two signaling pathways: JAK/STAT and mitogen-activated protein kinase (MAPK)/ERK (extracellular signal-regulated kinase) [8]. In the classical version, both pathways are activated uniformly and the subsequent evoked activation of inflammatory cytokine transcription is inhibited by the regulatory protein suppressor of cytokine signaling (SOCS) [8]. As a result of trans-signaling, there is a preferential activation of JAK/STAT and inhibition of signal transmission to SOCS, which contributes to the development of chronic inflammation [8].

Suppression of T cell death, inflammatory cell recruitment, and inhibition of regulatory T cell differentiation are among the pro-inflammatory activities of IL-6. One of the key molecular participants in acute phase reactions is IL-6, and there is a correlation between IL-6 and C-reactive protein (CRP) levels. Because of this, clinical practice uses both IL-6 and CRP as inflammatory indicators [9]. The anti-inflammatory effects of IL-6 in atherosclerosis include inhibition of the expression of pro-inflammatory cytokines: IL-1 and TNF-α, as well as increased expression of the anti-inflammatory cytokine IL-10 and increased synthesis of tissue inhibitor of matrix metalloproteinase (TIMP)-1, which promotes tissue regeneration [10]. It is obvious that IL-6 trans-signaling is dominant in atherosclerosis, which reflects the predominantly inflammatory role of IL-6. In particular, this may be due to the fact that IL-6R is expressed in a limited range of cells, such as some T cells, neutrophils and macrophages [8]. Despite this, it is important to understand the full picture of IL-6 interactions, which contributes to both a better understanding of atherosclerosis and the discovery of new potential therapeutic targets.

### 2.2 Tumor Necrosis Factor-Alpha (TNF-α)

One of the earliest expressed pro-inflammatory cytokines related to heart disease is TNF-α. Unlike IL-6, TNF-α has not yet been shown to have a dual nature of action in atherosclerosis: that is, it exhibits exclusively pro-inflammatory effects. Actually, TNF-α is produced by both macrophages and cardiac myocytes, and it can promote inflammation in both an autocrine and a paracrine manner [4]. This cytokine is also produced by T helper 1 (Th1) cells. There are two types of TNF-α: soluble and transmembrane. The synthesis of TNF-α triggers a series of pro-inflammatory transmitters that can affect tissue in a protective or damaging way. The very heterogeneous character of TNF-α signaling might be related to the fact that nearly every kind of cell has TNF-α receptors (TNFOR). For this cytokine, there are two primary receptor types: TNOR1 (p55) and TNOR2 (p75), which have different intracellular domains and hence trigger distinct signaling cascades. While TNOR2 stimulation is implicated in protective processes, TNOR1 activation is linked to negative outcomes [4]. It is interesting to note that TNF may shed its extracellular domains in response to specific stimuli, such as excess TNF-α in the bloodstream [11]. It is unclear, though, if this is an adaptive reaction to alleviate plasma TNF-α or if it actually increases TNF-α activity. Inducing cell death in both myocytes and endothelial cells is one of the main functions of TNF-α in the setting of cardiac tissue. Additionally, it plays a part in calcium dysregulation, attracts neutrophils and macrophages, and increases the generation of nitric oxide, all of which contribute to oxidative stress [12]. The desquamated soluble receptor TNF-PII is a significant endogenous regulator of TNF-α, and IL-10, an anti-inflammatory cytokine, inhibits TNF-α release. Furthermore, TNF-α has a role in thrombogenesis and angiogenesis, both of which are critical for the emergence of cardiac disease [13].

### 2.3 Interleukin-1 (IL-1) Family

In addition to TNF-α the most important inflammatory mediators in atherosclerosis are IL-1, IL-18 and IL-36 which are the three sub-families that make up the IL-1 family, which is composed of 11 ligands and 10 receptors [14]. The innate immune response is accompanied by the acute inflammation facilitated by the IL-1 family. This broad class of cytokines has aspects that both prevent and control inflammation, but it also primarily comprises pro-inflammatory components due to the large number of ligands and receptors associated [15].

IL-1α, IL-1β, and IL-18 are the three primary pro-inflammatory cytokines linked to the development of heart disease. In healthy options, epithelial and mesenchymal cells (including the heart) constitutively create IL-1α, which is released when the cell is damaged or died. In contrast, IL-1β is the major circulating type of IL-1 and is increased during disease. Like the previously examined cytokines, neutrophils, monocytes, and macrophages are the primary producers of IL-1β and IL-18. Prior to being triggered by NLRP3, nucleotide-binding domain, and caspase-1, they as well accumulate in the cytoplasm [16]. Primarily, IL-1β causes inflammation through the IL-6 signaling path and acute phase proteins such as CRP. However, IL-37 and IL-38, although also members of the IL-1 family, inhibit the levels of these pro-inflammatory cytokines. Moreover, the endogenously produced IL-1 receptor antagonist (IL-1Ra), also sold under the brand name anakinra, restricts the biological activities of IL-1α and IL-1β by binding to their receptor and blocking their connection [4].

### 2.4 Interferon-γ (IFN-γ)

IFN-γ, the only member of the type II IFN family, is linked to a number of heart diseases. This cytokine is produced by macrophages, CD4^+^ and CD8^+^ T lymphocytes, and natural killer (NK) cells and is involved in both innate and adaptive immunity [17]. IL-18 and IL-2 have the ability to promote IFN-γ production. IFN-γ causes signaling via the JAK/STAT pathway (just like the above-discussed IL-1, IL-6 and IL-18) when it is cleaved to its active state, which in turn promotes macrophages to accumulate low-density lipoprotein (LDL) [11]. Additionally, IFN-γ raises the expression of adhesion molecules on active endothelial cells and activates the expression of scavenger receptors on SMCs, facilitating their migration into the artery intima. IFN-γ promotes macrophage polarization, a pro-inflammatory M1 type that is also important in many cardiac diseases [17].

### 2.5 Granulocyte Colony-Stimulating Factor (G-CSF) and Granulocyte-Macrophage Colony-Stimulating Factor (GM-CSF)

In addition to the cytokines described above, which activate the effector functions of leukocytes, cytokines that affect the development and reproduction of cells involved in the pathogenesis are of great importance in the pathogenesis of atherosclerosis. Such cytokines include G-CSF and GM-CSF.

Human cardiomyocytes, monocytes, fibroblasts, and endothelial cells are the primary producers of G-CSF [11]. Its base function in the pathophysiology of the heart is to promote the growth and differentiation of neutrophils from monocytes. Moreover, G-CSF is found to protect vascular endothelial cells and cardiomyocytes from apoptosis [18].

T cells are the main producers of GM-CSF, although fibroblasts, endothelium and epithelial cells, and epithelial cells can also release it [11]. This cytokine promotes the survival, development, and propagation of neutrophils, eosinophils, macrophages, dendritic cells, and mast cells, among other functions that contribute to the initiation of inflammation [19].

### 2.6 Interleukin-2 (IL-2)

IL-2, like IL-6, despite its predominant pro-inflammatory functions, can exhibit a dual role in the pathogenesis of atherosclerosis. It has been demonstrated from experimental data that IL-2 has been associated with heart disease, despite not having received as much research as some other cytokines. It is well recognized that IL-2 plays a crucial role in the growth and survival of T regulatory cells, which are necessary for tolerance and immune response suppression [20]. But because it is critical for promoting effector T cell development and proliferation, IL-2 has a dual function in inflammation. Although it has mostly been employed in cancer clinical settings, it has been shown to have cardiotoxic effects, particularly at high dosages [21].

### 2.7 Interleukin-17 (IL-17)

Recently, scientists have started to comprehend the function of IL-17 in the pathophysiology of human disease. In comparison to other described cytokines, such as: IL-6, IL-1, IL-18, IFN-γ the role of IL-17 in the pathogenesis of atherosclerosis remains unclear. The primary source of IL-17 is a subset of CD4^+^ T helper cells known as Th17 cells, which produce large amounts of IL-17. Other T lymphocytes and myeloid cells have also been identified as having this ability [22]. The primary routes via which IL-17 functions are nuclear factor-κB (NF-κB) and MAPK, which result in the stability of target mRNA transcripts and the increase of pro-inflammatory gene transcription. IL-17 primarily targets non-hematopoietic cells, such as fibroblasts. The secretion of IL-17 can be induced by IL-18 [23].

## 3. Anti-Inflammatory Cytokines in the Pathogenesis of Atherosclerosis

### 3.1 Interleukin-5 (IL-5)/Interleukin-13 (IL-13)

Research using mice indicates that IL-5 and IL-13 may have an antiatherogenic effect [1]. It has been demonstrated that IL-5 increases the generation of neutralizing antibodies (IgM) to oxLDL, which in turn helps to shrink atherosclerotic plaques [24]. Research on the function of IL-13 in atherosclerosis has shown that recombinant IL-13 treatment stabilizes plaque by lowering macrophage accumulation, decreasing vascular adhesion molecule-1 (VCAM-1)-dependent monocyte enrollment, and raising collagen content [25]. Crucially, in *LDLr *^-⁣/-^ mice, IL-13 deficiency sped up the onset of atherosclerosis without changing blood cholesterol levels. As a result, IL-13 positively modifies plaque architecture and has preventive qualities against atherosclerosis [25].

### 3.2 Interleukin-27 (IL-27)/Interleukin-35 (IL-35)

Other cytokines that perform significant atheroprotective functions in addition to IL-5 and IL-13 are IL-27 and IL-35. A heterodimer made up of the subunits p28 and Ebi3, IL-27. The cytokines IL-27 and IL-35 have the same Ebi3 subunit [24]. With a wide range of effects on many cell types, IL-27 is considered as an anti-inflammatory cytokine [26]. Since IL-27 receptor-deficient animals show greater Th1 and Th17 CD4^+^ T cell activation and accumulation in the aorta as well as an increase in IL-17A and IL-17A-regulated chemokines (e.g., monocyte chemoattractant protein-1 (MCP-1)), IL-27 inhibits CD4^+^ T cell activation. This leads to an accumulation of different kinds of myeloid cells. Additionally, IL-27 suppresses the production of foam cells by preventing macrophages from accumulating lipids [27].

This heterodimer consists of the IL-35 subunits p35 and Ebi3. This anti-inflammatory cytokine originates from T-regulatory cells [24]. In addition to controlling the expression of anti-inflammatory cytokines, IL-35 limits the activation of CD4^+^ T-cells, promotes the development of T-regulatory cells, and delays the progression of inflammatory and autoimmune illnesses. The Ebi3 and p35 subunits have been identified as being present in the atherosclerotic aorta, and in mouse models predisposed to atherosclerosis, the loss of the Ebi3 subunit gene aggravates the disease [24]. According to study [28] IL-35 inhibits the MAPK signaling cascade, which in turn prevents endothelial cells from producing VCAM-1. This prevents acute inflammation in the vascular wall caused by lipopolysaccharide [28].

### 3.3 Interleukin-10 (IL-10)

Like IL-27 and IL-35, IL-10 signaling affects various immune cell types involved in the pathogenesis of atherosclerosis. The production of IL-10 by lymphocytes and macrophages (M2) is crucial for the regulation of both innate and adaptive immunity. The generation of IL-10 in lymphocytes is linked to a fraction of Th2, T regulatory cells, and, more recently, certain Th1 cells that produce IFN-γ. Atherosclerotic lesions, which are characterized by increased infiltration of inflammatory cells, particularly activated T cells, and elevated levels of pro-inflammatory cytokines, are promoted by IL-10 deficiency [29]. Leukocyte IL-10 appears to be crucial in controlling the cellular and collagen content of plaques as well as limiting the formation of atherosclerotic lesions [29]. Systemic or local overexpression of IL-10 by adenovirus gene transfer in generated carotid atherosclerosis in *LDLr *^-⁣/-^ mice was extremely efficient in avoiding atherosclerosis, which is consistent with the protective role of IL-10 in atherosclerosis. Interestingly, in *LDLr *^-⁣/-^ mice, the production of IL-10 by activated T cells decreased atherosclerosis, indicating a preventive effect against atherosclerosis [30].

### 3.4 Interleukin-19 (IL-19)

IL-19 is one of the most significant anti-inflammatory cytokines contributing to plaque healing. The IL-20R1 and IL-20R2 subunits together form a receptor complex via which IL-19 functions [31]. Monocytes, endothelial cells, fibroblasts, and CD8^+^ T cells are the main producers of IL-19. The function of SMCs, the formation of Th2-dependent immune responses, and the reduction of intimal hyperplasia during vascular wall inflammation are all regulated by IL-19 [31]. The activation of VSMCs and the generation of pro-inflammatory molecules including TNF-α, IL-1β, and MCP-1 are caused by IL-19 deficiency, according to recent research. Apart from stimulating VSMCs, IL-19 also regulates the activation of endothelial cells, as atherosclerosis-prone *IL19 *^-⁣/-^ mice produce more adhesion molecules. When considered collectively, these findings point to IL-19 as a strong inhibitor of the formation of atherosclerosis that regulates the migration, proliferation, and production of pro-inflammatory molecules in VSMCs [32].

### 3.5 Transforming Growth Factor-β (TGF-β)

TGF-β is essential for embryonic development because it controls cell division and proliferation. The natural structure of the blood vessel wall must be preserved [33]. Due to its ability to inhibit Th1 and Th2 cell proliferation, activation, and differentiation, TGF-β is also involved in immune cell regulation. Additionally, it is necessary for T regulatory cell differentiation [24]. TGF-β has antiatherogenic and anti-inflammatory properties in atherosclerosis. In *ApoE *^-⁣/-^ mice, inactivation or genetic ablation of TGF-β stimulates the development of atherosclerosis and makes it easier for pro-inflammatory macrophages and T lymphocytes to be recruited to the site of inflammation. TGF-β has also been demonstrated to lower the amount of collagen in the aorta. Consequently, TGF-β is an essential anti-atherogenic cytokine that is needed for the development of T-regulatory cells, which in turn inhibits effector T cells [34]. But despite being well-known for its anti-inflammatory qualities, TGF-β is a pleiotropic cytokine that contributes significantly to heart disease-related inflammation and cell damage. There are three isoforms of TGF-β, but TGF-β1 has been investigated in human physiology the most. While most heart cells may generate TGF-β, cardiomyocytes and invasive macrophages appear to be the primary causes of heart illness; in some situations, lymphocytes, induced fibrocytes, vascular endothelial cells, and mast cells might produce TGF-β [35]. TGF-β activity is closely related to T cell development. In response to IL-6, TGF-β promotes the development of T helper 17 (Th17), NK, and T regulatory cells [4]. TGF-β is a chemoattractant for neutrophils, monocytes, and other immune cells and aids in the polarization of macrophages towards the M2 phenotype [35]. As such, it plays a crucial role in the change from tissue inflammation to tissue healing.

**Fig. 1.**
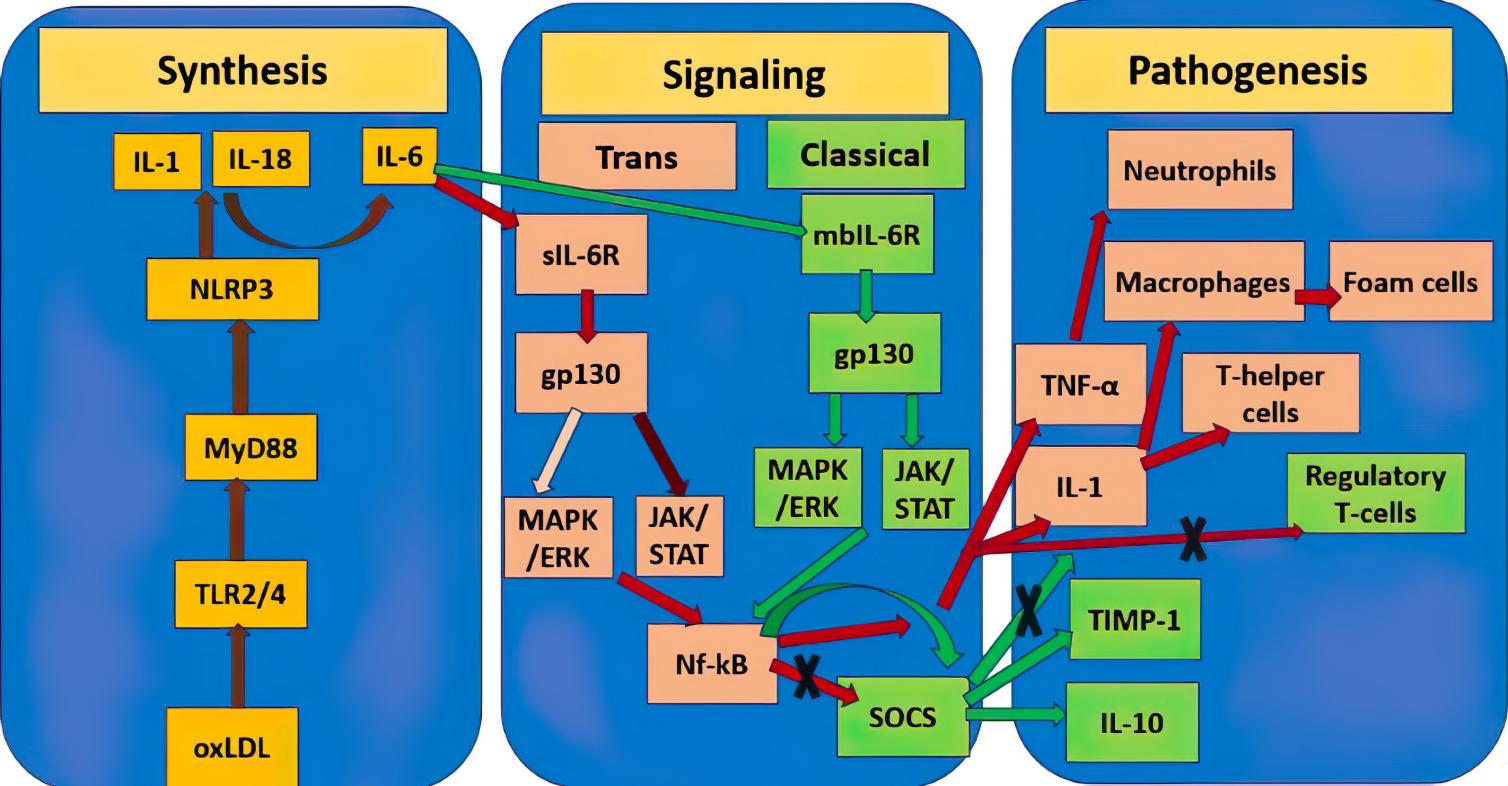
**Double role of IL-6 in the pathogenesis of atherosclerosis**. Yellow rectangles show mediators of the molecular pathway of initiation of atherosclerosis synthesis, the stages between them are connected by brown lines. Pink rectangles designate the atherogenic pro-inflammatory pathway triggered by IL-6, further stages are connected by red lines, the dark burgundy line designate a stronger signaling pathway relative to the weaker pale pink one. Green rectangles designate the atheroprotective anti-inflammatory pathway triggered by IL-6, further stages are connected by green lines. Crosses designate inhibition of this molecular pathway. TNF-α, tumor necrosis factor alfa; IL, interleukin; NLRP3, NOD-like receptor protein 3; MAPK, mitogen-activated protein kinase; TIMP, matrix metalloproteinase; gp130, glycoprotein 130; Nf-κB, nuclear factor-κB; sIL-6R, soluble IL-6 receptor; mbIL-6R, membrane-bound IL-6 receptor; MyD88, myeloid differentiation primary response gene 88; TLR, toll-like receptors; oxLDL, oxidized low density lipoprotein; JAK, Janus kinase; ERK, extracellular signal-regulated kinase; STAT, signal transducer and activator of transcription; SOCS, suppressor of cytokine signaling.

The paragraph above makes clear that, depending on a variety of parameters, including the kind of activation signal and the terms of the microenvironment, the same cytokines can have both pro- and anti-inflammatory characteristics. This is clear shown in Fig. 1 by example of IL-6. The significance of various cytokines in atherosclerosis is summarized in Table 1.

**Table 1.** **The importance of different forms of cytokines in the development of atherosclerosis**.

Cytokine	Inflammatory/atherogenic role in atherosclerosis	Anti-inflammatory/atheroprotective role in atherosclerosis
IL-6	∙ Initiation of IL-1 and TNF-α production	∙ Inhibition of TNF-α and IL-1 production
	∙ Recruitment of neutrophils/macrophages	∙ Initiation of IL-10 production
	∙ Inhibition of T helper cell apoptosis	∙ Initiation of TIMP-1 production
	∙ Inhibition of regulatory T cell differentiation	
	∙ Induction of foam cell formation	
TNF-α	∙ Induction of apoptosis in endothelial cells	-
	∙ Recruitment of neutrophils/macrophages	
	∙ Induction of oxidative stress	
IL-1β/IL-18	∙ Induction of the acute phase of inflammation	-
	∙ Recruitment of neutrophils/macrophages	
IL-37/IL-38	-	∙ Reduction of IL-1β levels
IFN-γ	∙ Induction of foam cell formation	-
	∙ Activation of macrophage polarization into M1 phenotype	
	∙ Increased expression of adhesion molecules in the endothelium	
TGF-β	∙ Activation of differentiation of NK and Th-17 lymphocytes	∙ Activation of T-regulatory cell differentiation
		∙ Activation of macrophage polarization into M2 phenotype
IL-5		∙ Initiation of IgM secretion to LDL
IL-13		∙ Decreased macrophage accumulation
		∙ Plague stabilisation
IL-27/IL-35		∙ Inhibition CD4^+^ T cells activation
		∙ Inhibition of foam cells formation
		∙ Promoting the development of T-regulatory cells
IL-10	-	∙ Inhibition of inflammatory gene activation
IL-19		∙ Intimal hyperplasia
		∙ Decreasing of endothelial cell activation
		∙ Inhibition of IL-1β and TNF-α production

Abbreviations: IL, interleukin; TNF-α, tumor necrosis factor alfa; IFN-γ, interferon gamma; TGF-β, transforming growth factor beta; TIMP, matrix metalloproteinase; LDL, low-density lipoprotein; NK, natural killer.

## 4. Targeted Therapy Aimed at Inhibiting Pro-Inflammatory Cytokines

### 4.1 Impact on IL-1 Signaling Pathway

The subject of atherosclerotic cardiovascular disease has greatly benefited from recent studies [36, 37]. A significant phase 3 clinical study has shown for the first time that patients with stable atherosclerosis can benefit clinically from therapeutic targeting of the inflammatory response [38]. The goal of the investigation was to assess the impact of canakinumab, a monoclonal antibody that targets IL-1β. Every three months, patients, the majority of whom were on statin medication, were given three different dosages of either placebo or canakinumab: 50 mg, 150 mg, or 300 mg. The middle dosage (150 mg) produced the greatest outcomes, reducing the main destination of nonfatal myocardial infarction, nonfatal stroke, or cardiovascular mortality by 15%. Nevertheless, the suppression of IL-1β also markedly increased the rate of fatal infections, as it is also a crucial cytokine in host defences against bacterial infection. This study clearly shows the positive benefit of blocking IL-1β signaling, even with these notable adverse effects [38].

As an IL-1 receptor antagonist, anakinra inhibits both the effect of IL-1α and IL-1β isoforms. Patients with rheumatoid arthritis, who are also identified to be at high risk of cardiovascular disease, have been treated with it since 2001 [39]. Small-scale clinical studies involving myocardial infarction patients have assessed anakinra. This kind of interleukin-1 blocking was found to lower the incidence of heart failure and to dramatically lower the systemic inflammatory response in myocardial infarction patients, as well as to strongly lower the levels of high-sensitivity C-reactive protein (hsCRP). In a different trial, individuals who took Anakinra daily for two weeks also showed decreased levels of hsCRP and IL-6. On the other hand, by day 30, the patients on Anakinra had far higher hsCRP levels than the patients on placebo, and there was an unexpected rise in late repetitive ischemic episodes.

In conclusion, it seems that there is more distinction to the link between IL-1 targeting and clinical results than first believed. Medications designed to target IL-1β (Canakinumab) or both IL-1α and IL-1β with human recombinant IL-1RA (Anakinra) are among the therapeutic approaches that are now being trialled [34]. Blocking other molecular targets that trigger the inflammatory response at earlier stages – those responsible for signaling maturation and IL-1 synthesis – has potential prospects. For example, a study [40] showed that blocking myeloid differentiation primary response gene 88 (MyD88), a mediator responsible for the initial stages of innate immunity after TLR signaling, by administering the small molecule inhibitor LM9 reduced oxidative stress, vascular inflammation and foam cell formation in *ApoE *^-⁣/-^ mice. Moreover, the main cells with increased MyD88 production were infiltrated macrophages, which demonstrates the targeted effect of LM9 on pathogenic cells without affecting normal cells. In future clinical trials, this could potentially contribute to fewer side effects from LM9 administration. Another example is the compound MCC950, which selectively inhibits NLRP3 in macrophages. In an atherosclerotic model of *ApoE *^-⁣/-^, it was shown that administration of this compound reduced plaque size and the number of macrophages, and also reduced the production of IL-1β and IL-18 [41].

### 4.2 Effect on Tumor Necrosis Factor (TNF)

Currently, anti-TNF monoclonal antibodies (adalimumab, infliximab, golimumab, and certolizumab pegol) and soluble TNF receptor (etanercept) are the five anti-TNF medications authorized for clinical usage. These are accepted for the treatment of psoriatic arthritis, ulcerative colitis, rheumatoid arthritis, and ankylosing spondylitis, golimumab is a completely humanized anti-TNF monoclonal antibody.

Despite the lack of information on the therapeutic effectiveness of golimumab in atherosclerosis, positive findings from a pilot research were reported [42]. The purpose of this double-blind, randomized, placebo-controlled trial was to determine how well golimumab works in individuals with ankylosing spondylitis (AS) to slow down the development of arterial stiffness and atherosclerosis. 20 patients received monthly golimumab dosages of 50 mg, whereas 21 individuals received a placebo for a full year. Vascular measures (such as aortic stiffness and carotid intima/media thickness) did not significantly differ between the two groups after six months. On the other hand, only the placebo group showed a significant increase in mean intima media thickness (IMT) compared to the golimumab group. The augmentation index (Aix), maximum IMT, and pulse wave velocity (PWV) did not alter. After a year of therapy, there were no appreciable variations in vascular markers between the two groups.

Additional extensive research is required to thoroughly examine the possible impacts noted in this investigation. TNF inhibitors have also been shown to strengthen the overall pathological characteristics with rheumatoid arthritis (RA) and psoriasis who are at high cardiovascular risk, in addition to these findings in atherosclerosis patients. TNF targeting therapy has been beneficial in avoiding atherosclerosis in RA, and it is possible that this is also the case in psoriasis [43]. The demonstrated efficacy of TNF inhibitors for the treatment of other inflammatory diseases provides a good basis for using existing developments for testing directly on atherosclerosis models. For example, a study [44] showed that ezetimibe contributed to a decrease in serum cholesterol levels, a decrease in the concentration of inflammatory cytokines (MCP-1 and TNF-α) and inhibition of macrophage accumulation in lesions in *ApoE *^-⁣/-^ mice both in single therapy and in combination with atorvastatin. In addition, the discovery of new medicinal compounds that block the action of TNF-α is also important. The compound tanshinone IIA, a plant diterpene, has been shown to have an atheroprotective effect on HUVEC cell culture, which consists of reducing the expression of adhesion chemokines VCAM-1, intercellular adhesion molecule-1 (ICAM-1) and C-X3-C motif chemokine ligand 1 (CX3CL1) through the suppression of TNF-α signaling [45]. In addition, the use of tanshinone IIA did not reveal a cytotoxic effect, which is a favorable basis for assessing safety in future clinical trials.

### 4.3 Broad-Spectrum Anti-Inflammatory Drugs

Low-dose methotrexate (LD-MTX) treatment reduces circulating levels of CRP, IL-6, TNF-alpha, and cardiovascular events in patients with RA. An important feature of LD-MTX is its ability to increase adenosine production and stimulate the adenosine A2A receptor, which has been shown to promote the expression of several proteins involved in reverse cholesterol transport, thereby potentially reducing foam cell formation [46]. However, one should be careful when choosing doses and the mode of administration of such a systemic drug. Thus, in a clinical study [47], methotrexate, even in low doses (15 to 20 mg per week) when administered to patients with myocardial infarction and type 2 diabetes, was associated with a higher risk of developing severe side effects, including the development of non-basal cell skin cancer. In addition, it did not show effectiveness in reducing inflammation in patients with atherosclerosis—the levels of IL-1β, IL-6 and CRP did not decrease.

Colchicine is a cheap anti-inflammatory medication that is prescribed to people with pericarditis, familial Mediterranean fever, and gout. Colchicine may disrupt phagocytosis, inflammasome activation, microtubule-based inflammatory cell chemotaxis, and other host immunological processes by blocking microtubule assembly. The initial benefit of colchicine on coronary artery disease (CAD) was noted in individuals who had a family history of Mediterranean fever [43]. Consequently, in order to assess the safety and effectiveness of long-term low-dose colchicine therapy in patients with acute coronary syndrome, the low dose colchicine (LoDoCo) research was created as a prospective trial. Randomization was used to assign 532 individuals receiving antithrombotic treatment and lipid-lowering medication to receive either no colchicine or a daily dosage of 0.5 mg/mL. Compared to 16% in the no-treatment group, 5.3% of patients in the low-dose colchicine group experienced the main endpoint (acute coronary syndrome, out-of-hospital cardiac arrest, or non-cardioembolic ischemic stroke) after a median follow-up of three years [48].

### 4.4 Approaches Seeking the Balance Regulatory and Effector T Cells

While there aren’t any T cell-targeted treatments being used in clinical care medicine right now to treat or prevent cardiovascular disease, a number of therapeutic strategies are being tested in clinical trials after showing promise in preclinical models. It has been demonstrated that regulatory T lymphocytes aid in the development and stagnation of atherosclerosis in mice that are susceptible to the condition [49]. On the other hand, many effector T cells—particularly the Th1 cells that secrete IFNγ—are thought to be proatherogenic [50].

Therefore, the aim of T cell-targeted treatment methods in atherosclerosis is to modify the homeostatic balance of various T cell subsets by reducing populations of supposedly proatherogenic effector T cells and increasing atheroprotective, immunosuppressive T regulatory cells [51]. One approach to do this is by targeting pathways like IL-2 and boosting non-specific T regulatory cells. However, tolerogenic vaccinations against atherosclerosis-related antigens can be used to increase the number of certain T regulatory cells.

### 4.5 Effect on IL-6

For the monoclonal antibody ziltivekimab, directed against IL-6, in 2 clinical phase 2 studies: RESCUE (in patients from the USA) [52] and RESCUE-2 [53] (in patients from Japan) demonstrated the effectiveness in reducing inflammatory and thrombotic markers of atherosclerosis in patients with chronic kidney disease, at high risk of developing atherosclerosis. According to the results of the studies, the levels of CRP in the groups of patients receiving ziltivekimab decreased in a dose-dependent manner from 77 to 96% compared with 4% (RESCUE) and 27% (RESCUE-2) in the placebo groups. There was also a significant decrease in such markers as: fibrinogen, serum amyloid A, haptoglobin, secretory phospholipase A2 and lipoprotein A. No significant side effects were observed. The success of these studies has allowed preparations to begin for a phase 3 clinical trial of ziltivekimab (ZEUS) [54] in a larger sample of patients with chronic kidney disease at high risk of developing atherosclerosis.

Given the dual nature of IL-6 in the development of atherosclerosis, it makes sense to study other IL-6 signaling mediators as targets, which determine whether the IL-6 signal will go via the classical or trans-pathway. Thus, it was shown that the IL-6R Asp358Ala variant, common in atherosclerosis, defectively binds to the leukocyte membrane, which blocks the classical transmission of the IL-6 signal [55]. Enhancement of the function of SOCS, a mediator that acts as a feedback regulator, blocking the inflammatory response of IL-6, may have great potential. Thus, the review considers the possibility of using SOCS mimetic drugs for the treatment of autoimmune diseases [56], taking into account the analysis carried out in this review, we can also assume with great confidence the potential for using these mimetics in the treatment of atherosclerosis.

The clinical studies described in this section are summarized in Table 2.

**Table 2.** **Clinical trials investigating the effects of drugs on inflammatory targets in atherosclerosis**.

Drug	Target	Clinical trial	Results
Canakinumab	IL-1β	III phase	Reduction in cardiovascular events, but development of infectious complications.
Anakira	IL-1α and IL-1β	Pilot study	No definitive reduction in inflammatory mediators.
Golimumab	TNF-α	Pilot study	No therapeutic effect was shown.
Methotrexate	Dihydrofolate reductase	III phase	Severe side effects. There was no therapeutic effect in inflammatory mediators decline.
Colchicine	NLRP3	Pilot study (LoDoCo)	Reduced risk of cardiovascular events
Ziltivekimab	IL-6	II phase (RESCUE and RESCUE-2)	Significant decrease in biomarkers of atherosclerosis. No significant side effects were shown.

Abbreviations: LoDoCo, low dose colchicine; TNF-α, tumor necrosis factor alfa; IL, interleukin; NLRP3, NOD-like receptor protein 3.

## 5. Discussion

There is presently insufficient data to support the beneficial effects of targeted anti-inflammatory medication in the treatment of atherosclerosis, despite the complexity and imperfect understanding of immune and inflammatory networks. This is true even if our understanding of the underlying mechanisms of cytokine activity in humans is continually growing. Specifically, as evidenced by the MRC-ILA-HEART study [57] and more direct and indirect anti-inflammatory approaches, these therapies may in some circumstances even raise the risk of unfavorable cardiovascular events. In fact, recent research indicates that several medications showing promise in animals do not work as well in people. Agents against IL-17 and IL-12/23p40 are two examples. A recent meta-analysis shown that therapy with such medicines (bikanumab and estekinumab) may even increase the risk of significant adverse cardiovascular events when compared to placebo, despite their proatherogenic effects in animals [58]. Due to the pro-inflammatory properties of IL-17, ixekizumab, secukinumab, and brodalumab—all IL-17 receptor A antagonists can potentially block atherosclerosis. These drugs are also used to treat psoriasis. IL-17 inhibition reduces atherosclerosis in animals, although there are currently no human clinical trials available [59].

Other research, however, has demonstrated that these anti-inflammatory techniques offer important advantages. All things considered, there seems to be a big disconnect between the advantages of such focused therapies and our comprehensive understanding of cytokine activity. This might be explained by the many cytokines involved in atherogenesis and their complicated effects, as well as the shortage of large-scale clinical trials that could also offer trustworthy information on the effectiveness of these theories. Therefore, cytokine modulation poses a therapeutic conundrum, and treatment must take into account the advantages and disadvantages of reducing inflammation. Observations are mostly restricted to rheumatic patients, especially for novel biologic treatments, and data demonstrating the benefits of some settings (e.g., TNF inhibitors) for survival and health is weak in small-scale clinical studies. It is interesting to note that there are a number of these clinical studies now taking place (most notably the CANTOS, CIRT, and Entracte trials), the outcomes of which are widely anticipated and might influence the course of atherosclerosis research in the future [60, 61, 62]. In addition, a promising future direction is the development and testing of mimetics of anti-inflammatory cytokines with a wide range of functions (IL-27, IL-35, IL-19). Another important direction is the study of the mechanisms of “switching” from atherogenic to atheroprotective action for cytokines that show a dual role in atherosclerosis: IL-6, TGF-β.

## 6. Conclusions

Both pro- and anti-inflammatory cytokines play a major role in the development of atherosclerosis. Some cytokines can play both of these roles to a greater or lesser extent, for example: IL-6 and TGF-β, which creates prerequisites for further study into the pathogenesis of atherosclerosis. The most promising therapeutic target for the treatment of atherosclerosis among cytokines is IL-6: the antibody ziltivekimab developed against it has shown efficacy and safety in 2 phase II clinical trials. There are also several promising therapeutic targets for which therapeutic efficacy has already been demonstrated in preclinical studies.
